# Injectable Magnesium-Zinc Alloy Containing Hydrogel Complex for Bone Regeneration

**DOI:** 10.3389/fbioe.2020.617585

**Published:** 2020-11-26

**Authors:** Wei-Hua Wang, Fei Wang, Hai-Feng Zhao, Ke Yan, Cui-Ling Huang, Yin Yin, Qiang Huang, Zao-Zao Chen, Wen-Yu Zhu

**Affiliations:** ^1^Department of Neurosurgery, The Affiliated Suzhou Science and Technology Town Hospital, Nanjing Medical University, Suzhou, China; ^2^Institute of Clinical Medicine Research, The Affiliated Suzhou Science and Technology Town Hospital, Nanjing Medical University, Suzhou, China; ^3^Institute of Biomaterials and Medical Devices, Southeast University, Suzhou, China; ^4^Department of Pathology, The Affiliated Suzhou Science and Technology Town Hospital, Nanjing Medical University, Suzhou, China; ^5^Department of Neurology, The Second People’s Hospital of Longgang District, Shenzhen, China; ^6^Laboratory Animal Center, Soochow University, Suzhou, China; ^7^Department of Neurosurgery, The Second Affiliated Hospital, Soochow University, Suzhou, China; ^8^School of Biological Science and Medical Engineering, Southeast University, Nanjing, China

**Keywords:** gelatin methacryloyl, hydrogel, magnesium–zinc alloy, calvarial defect, bone regeneration

## Abstract

Gelatin methacryloyl (GelMA) has been widely used in bone engineering. It can also be filled into the calvarial defects with irregular shape. However, lack of osteoinductive capacity limits its potential as a candidate repair material for calvarial defects. In this study, we developed an injectable magnesium–zinc alloy containing hydrogel complex (Mg-IHC), in which the alloy was fabricated in an atomization process and had small sphere, regular shape, and good fluidity. Mg-IHC can be injected and plastically shaped. After cross-linking, it contents the elastic modulus similar to GelMA, and has inner holes suitable for nutrient transportation. Furthermore, Mg-IHC showed promising biocompatibility according to our evaluations of its cell adhesion, growth status, and proliferating activity. The results of alkaline phosphatase (ALP) activity, ALP staining, alizarin red staining, and real-time polymerase chain reaction (PCR) further indicated that Mg-IHC could significantly promote the osteogenic differentiation of MC3T3-E1 cells and upregulate the genetic expression of collagen I (COL-I), osteocalcin (OCN), and runt-related transcription factor 2 (RUNX2). Finally, after applied to a mouse model of critical-sized calvarial defect, Mg-IHC remarkably enhanced bone formation at the defect site. All of these results suggest that Mg-IHC can promote bone regeneration and can be potentially considered as a candidate for calvarial defect repairing.

## Introduction

Autologous skull graft is generally considered as the gold standard for cranioplasty (Lucateli et al., 2018). However, it usually has high probability of bone resorption which may cause bone defects and impair reimplantation (Chan et al., 2017; Barzaghi et al., 2019). In our previous study, varying degrees of bone defects were found due to bone resorption after calvarial reimplantation in Beagle dogs, which were very similar to what happened clinically (Zhu et al., 2020). In addition, congenital hypoplasia, chronic infections, or bone tumors may also cause calvarial bone defects. Different from the long bones, once the adult’s skull is defective, it is difficult to repair and regenerate spontaneously (Burdette et al., 2018). Therefore, it is necessary to pursue appropriate treatments to repair calvarial defects, where bone repair materials could play a big role.

Hydrogel is a kind of insoluble hydrophilic polymer, whose weight will increase to several times of its original dry weight after soaked in water. Since its internal structure is similar to the extracellular matrix of tissue, where nutrients and metabolites are able to be transported, hydrogel has been widely used in tissue engineering (Seliktar, 2012). So far, various kinds of natural and synthetic hydrogel polymer have been developed, including gelatin, alginate, fibrin, chitosan, hyaluronic acid (HA), poly (ethylene oxide) (PEO), and poly (ethylene glycol) (PEG) (Vega et al., 2017). Among them, gelatin methacryloyl (GelMA), which is made of gelatin and methacrylic anhydride has been paid increasingly attention in their usage in bone tissue engineering research and clinics. GelMA has been demonstrated promising biocompatibility and biodegradability, while very low cytotoxicity and immunogenicity (Yue et al., 2015). GelMA contains arginine-glycine-aspartic acid (RGD) peptide sequence which promotes cell adhesion, proliferation, and differentiation (Liu and Chan-Park, 2010; Wohlrab et al., 2012; Qiao et al., 2020). Moreover, because of the presence of methacrylic anhydride, GelMA is able to generate a cross-linked hydrogel with thermal stability by being applied photoinitiator and under ultraviolet (UV) irradiation. With this characteristic, the aqueous solution of GelMA can be injected into irregularly shaped bone defects and get solidified afterward (Annabi et al., 2014). As a result, GelMA has been applied as bone regeneration scaffolds for bone repair in recent researches (Kwon et al., 2018; Gu et al., 2019; Pan et al., 2019). However, due to the lack of osteoinductive capacity essential for bone mineralization, GelMA are often applied in conjunction with other materials such as inorganic particles, biopolymers, nanomaterials, and so on.

Magnesium and its alloy materials have been widely used as bone repair materials (Agarwal et al., 2016). Magnesium ion is the second most abundant intracellular cation in mammals, and more than half of which is stored in bone (Weisinger and Bellorin-Font, 1998; Wolf and Cittadini, 2003). Compared with other metals, magnesium and its alloys have several obvious advantages in bone repair. First, the density and elastic modulus of magnesium are close to those of natural bone; second, magnesium can be degraded *in vivo* and mainly release non-toxic magnesium oxide and Mg^2+^ during degradation, which can be completely excreted through urine (Saris et al., 2000; Nagels et al., 2003; Paschoal et al., 2003; Yu et al., 2018). More importantly, many evidences demonstrated that applications of magnesium *in vivo* could inhibit bacterial growth and promote new bone growth (Yamasaki et al., 2002; Li et al., 2014). Since the application of pure magnesium may lead to emphysema, etc., recent studies have focused on magnesium alloys which can improve the mechanical properties, osteoinductive capacity, the stability of the material, and decrease the degradation and its reactivity *in vivo*. However, the uncontrollable degradation rate of magnesium alloys impedes their applications in clinic. If degenerated too fast, it may lose its function before the bone heals completely, while lower degradation rate leads to negative effects on early bone healing. In addition, the shapes of calvarial defects caused by bone resorption are always irregular and changing over time, so the “intramedullary nail” used in long bones, or the screws, etc., are unable to be applied (Wang et al., 2017). Therefore, there is an unmet need to develop plastic magnesium alloys with promising degradability, especially for calvarial defects.

In this study, we report our development of an injectable hydrogel complex with GelMA and biodegradable magnesium–zinc alloy [injectable magnesium–zinc alloy containing hydrogel complex (Mg-IHC)]. The mechanical properties, plasticity and biocompatibility, of Mg-IHC were evaluated as well as its effect on osteogenis. Furthermore, we applied Mg-IHC on a mouse model of critical-sized calvarial defect to investigate its efficacy on bone regeneration. Mg-IHC was filled into the skull defect, photo cross-linked, and sustained release of magnesium. Our result demonstrated its effectiveness in promotion of the osteogeneis and the reparation of calvarial defects.

## Materials and Methods

### Preparation of GelMA and Mg-IHC

Gelatin (Sigma, United States) with the amount of 10 g was stirred until completely dissolved in 100 mL of carbonate buffer (pH 8) at 50°C. Then, 10 mL methacrylic anhydride was slowly added and stirred at 50°C for 2 h to make the gelatin fully react with methacrylic anhydride (Sigma, United States) which was terminated by dilution with 400 mL of double-distilled water (DD water) at 40°C. The reaction solution was dialysis at 37°C for 7 days, with the DD water changed every 4 h. Finally, the dialysate was lyophilized to obtain a white solid product, which was maintained at −80°C. The degree of methacrylation was determined as 95% by ^1^H NMR. The GelMA solid was mixed and placed at 37°C more than half an hour until the solid completely dissolved. GelMA prepolymer was prepared in the final concentration of 10% (w/v) with 0.5% (w/v) photoinitiator (LAP) (Sigma, United States).

In order to pass through the syringe needle smoothly after mixing, the biomedical magnesium (95%, wt%) zinc (5%, wt%) alloy (Weihao, China) was fabricated into circular microspheres with the diameter of 20 ± 10 μm. In order to obtain spherical microspheres with high purity and promising fluidity, processes such as inert gas protection, rapid cooling, and atomization were applied.

Before use, Mg-IHC solution was prepared by homogeneous with GelMA composite of Mg–Zn alloy of 0.5, 1, 2, 3, and 5% with the mass volume ratio.

### Measurement of Elastic Modulus of GelMA and Mg-IHC

Gelatin methacryloyl and Mg-IHC prepolymer solution were injected into the custom molds (diameter 10 mm; thickness 1 mm), then exposed to UV light for 1 min at 25 mW/cm^2^ power. The elastic modulus of the hydrogel was characterized by nano-indenter (PIUMA, Optics11, Netherlands). For analysis, we selected an appropriate probe with stiffness value = 0.5 N/m, tip radius value = 50 μm.

### Scanning Electron Microscope (SEM)

The cross-linked GelMA or Mg-IHC (1% alloy containing) were quickly frozen in liquid nitrogen and then lyophilized in a lyophilizer (Toshiba, Japan). The samples were placed on the sample table and sputter coated with gold for 40 s and then the hydrogel pore size images were taken at 5 kV with a scanning electron microscope (PHENOM ProX SEM, Phenom-China).

### Material Degradation

For degradation assay, 500 μL of GelMA and Mg-IHC were exposed to UV light for 1 min to crosslink and original weights were recorded and then added into 10 mL PBS. They were then incubated at 37°C on a shaker at 50 r/min, and weighed as the later weight at day 1, 3, 7, 14, 21, and 28, respectively. The degradation rate was calculated according to the formula: degradation rate = (original weight − later weight)/original weight × 100%.

### Magnesium Ion Releasing

In order to detect the magnesium release from Mg-IHC, 100 μL Mg-IHC with various magnesium alloys was used. With irradiation under UV light for 1 min, Mg-IHC were cultured with 5 mL α-MEM at 37°C for 3, 7, 14, 21, and 28 days. Magnesium ion concentration was detected at each time point using the Magnesium Ion Concentration Detection Kit (Leagene, China).

### Cell Culture, Cell Adhesion, Viability, and Proliferation

Mouse osteoblast MC3T3-E1 cells were cultured with the medium [α-MEM and 10% fetal bovine serum (FBS) plus 1% penicillin-streptomycin (P/S)]. Leaching solutions were prepared as below. 100 μL GelMA or Mg-IHC were injected into the 6-cm dish and solidified by illumination with the UV light. Then 5 mL culture medium was added and incubated for 24h at 37°C to make a leaching solution.

For cell proliferation, cells were seeded in 96-well plate with 2 × 10^4^ in each well and evaluated with CCK-8 kit (BBI, China). 100 μL leaching solutions were added with the culture medium as the control. After 1, 3, and 7 days, absorbance was measured at 450 nm using a microplate reader (Tecan Infinite 200 pro, United States).

In order to examine the cell viability, cells were seeded in 24-well plate at a density of 2 × 10^4^ per well. Leaching solutions were also used as the culture medium. Calcein and propidium iodide (PI) double staining were performed with live/dead staining assay kit (Beyotime, China). Images were captured on a fluorescence microscope (Olympus BX 53, Japan). And cells with green and red fluorescence were counted by ImageJ.

For cell adhesion assay, GelMA or Mg-IHC were coated on the bottoms of 24-well plate. Cells were cultured in the plate for 24 h, fixed and stained with FITC-Phalloidin (100 nM, Thermo Fisher Scientific, United States) for 30 min and with DAPI (BBI, China) to label the cellular nuclei for 10 min. Images were captured on a fluorescence microscope (Olympus BX 53, Japan).

### ALP and Alizarin Red Assay

In order to examine the effect of Mg-IHC on osteogenesis, alkaline phosphatase (ALP) and alizarin red staining were performed; meanwhile, ALP activity was also detected. The MC3T3-E1 cells were seeded in 24-well plate with 2 × 10^4^ each well. Leaching solutions were applied for cell culture with supplement of ascorbic acid (50 μg/mL) and β-glycerophosphate (10 mM). ALP staining and activity were conducted as the ALP staining kit (Solarbio, China) and ALP activity kit (Beyotime, China) at 7 days post treatment. To normalize the quantification, total protein concentration was measured by BCA assay kit (Beyotime, China). Alizarin red staining was conducted at 14 days post treatment. Absorbance was measured at 490 nm by the microplate spectrophotometer (Tecan Infinite 200 pro, United States). Images of staining were obtained by an Olympus BX53 microscope.

### Real-Time PCR Analysis

MC3T3-E1 cells were cultured with osteoinduction as above. Total RNA was extracted at 7 days post treatment with Trizol regent (Thermo Fisher, United States) according to the manufacturer’s protocol. Subsequently, reverse transcription was conducted using the First-Strand Synthesis System (Thermo Fisher, United States). Real-time polymerase chain reaction (PCR) (Thermo Fisher, United States) was then performed and β-actin was used as the housekeeping control. Quantitative data were performed by comparative Ct quantification (2^–ΔΔCt^ method). The primer sequences are listed in Table 1.

**TABLE 1:** Sequences of primers used for real-time PCR.

Genes	Primers	Sequences (5′-3′)
COL1	Forward	GCTCCTCTTAGGGGCCACT
	Reverse	CCACGTCTCACCATTGGGG
OCN	Forward	CTTGAAGACCGCCTACAAAC
	Reverse	GCTGCTGTGACATCCATAC
RUNX2	Forward	CCGTGGCCTTCAAGGTTGT
	Reverse	TTCATAACAGCGGAGGCATTT
β-actin	Forward	CTGACTGACTACCTC
	Reverse	GACAGCGAGGCCAGGATG

### Animal Model of Critical-Sized Calvarial Defect

Animals used in this study were handled according to the protocols approved by the Institutional Animal Care and Use Committee of Soochow University.

Eight-week-old female ICR mice were used in this study. After anesthesia, the scalp was cut along the midline of parietal lobe to expose the skull. Craniectomy with 3.5 mm defects were trephinated on the center of both side of midline of all animals with a special skull drill (Huamai, China). GelMA or Mg-IHC was injected in the right defect. UV exposure was used for 1 min to solidify the hydrogels. The defects on the left side were without treatment and set as the control.

### Micro-Computed Tomography Analysis

Four weeks after surgery, mice were sacrificed to harvest the calvarias for micro-computed tomography (micro-CT). In brief, the bones were dissected out and cleaned the soft tissue as much as possible. After fixed in 10% formalin for at least 12 h, the calvarias were scanned with the micro-CT (SkyScan1176, Belgium) with the following settings: 50 kV, 500 mA, and 0.5 mm Al filter. Regions of interesting (ROI) on calvarial defects of 3.5 mm in diameter were chosen to analyze the parameters of bone volume fraction [bone volume/tissue volume (BV/TV)], and bone mineral densities (BMDs).

### Histological Analysis

Calvaria bone samples were decalcified in 0.25 M EDTA (pH 7.4) for 10 days and then immersed in PBS for 2 h. After dehydration in gradient alcohol (70, 80, 90, 95, and 100%), samples were embedded in paraffin and sectioned at the thickness of 5 μm. Sections were stain with hematoxylin and eosin (H&E), Masson staining (Solarbio, China) and imaged using a lighting microscope (BX43, OLYMPUS, Japan). Masson sections (*n* = 5 for each group) were analyzed for the total tissue area and mature bone area of the defects. The kidney and liver of the mice were also harvested for H&E staining as the procedure above.

### Statistical Analysis

All statistical analysis was performed using SPSS20.0. Data are presented as the mean ± SEM. Data between multiple groups were analyzed by one-way or two-way analysis of variance (ANOVA) followed by Fischer’s PLSD *post hoc* tests. Comparisons between two groups were made by Student’s two-tailed unpaired *T*-test. *P* < 0.05 was considered for the significance level for all analyses.

## Results and Discussion

### Characterization of Injectable Magnesium–Zinc Alloy Containing Hydrogel Complex

In this study, we developed an injectable magnesium alloy hydrogel (Mg-IHC), which was uniformly mixed by Mg–Zn alloy microspheres and GelMA according to a certain proportion. The magnesium alloy microspheres were fabricated through a rapid cooling and atomization process with the protection of inert gas. The contents of magnesium and zinc were 95% and 5% (w/w), respectively. Magnesium and zinc are both fundamental elements in human bodies. It was reported that magnesium alloys containing about 5.0% zinc have excellent strength, high corrosion resistance, low hydrogen evolution rate, and promising animal biocompatibility (Zberg et al., 2009).

**FIGURE 1:**
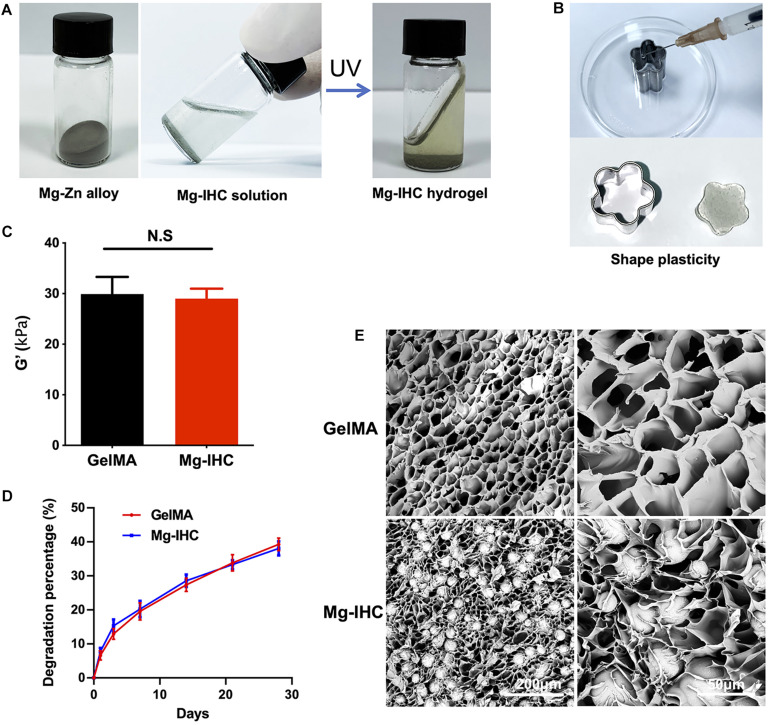
Structure and mechanical properties of Mg-IHC. **(A)** Schematic illustrations of Mg-IHC formed after UV irradiation. **(B)** Schematic of Mg-IHC molding. **(C)** Elastic modulus of GelMA and Mg-IHC. **(D)** Degradation percentage of GelMA and Mg-IHC *in vitro*. **(E)** Scanning electron microscopy imaging of GelMA and Mg-IHC.

The diameter of the magnesium alloy microspheres used in this research was 20 ± 10 μm, which was significantly smaller than the inner diameter of the syringe needle commonly used in clinic, to ensure the Mg-IHC could be injected freely into the calvarial defects. After application of UV irradiation for 1 min, Mg-IHC crosslinked to form a stable shape implied that it has good plasticity to be filled into the calvarial defects with irregular shape (Figures 1A,B). In addition, we measured the elastic modulus to evaluate the mechanical properties of GelMA and Mg-IHC. The result showed that Mg-IHC had similar elastic modulus to GelMA (Figure 1C).

The stiffness of the GelMA enhances as the degree of methacrylation increase, which results in an increase in the mechanical properties (Yue et al., 2015). In this research, we chose the high degree of methacrylation GelMA with high elasticity moduli and low viscosity.

Hydrogel degradation is an important property for determining stability under physiologically relevant conditions. To evaluate the degradation of GelMA and Mg-IHC *in vitro*, we immersed the same mass of cured composite hydrogel into the same volume of PBS, and weighed the remaining material at different time points. Both GelMA and Mg-IHC decreased in quality over time, and about 60% of the total weight remained after 4 weeks. Moreover, there was no statistical difference in the degradation rate of Mg-IHC at each time point (Figure 1D), which indicated that the mixture of Mg–Zn alloy microspheres would not affect the degradation of GelMA.

Appropriate internal porosity is necessary for tissue engineering materials to maintain cell growth and nutrient transfer (Karageorgiou and Kaplan, 2005). Therefore, we monitored the porosity of the GelMA and Mg-IHC hydrogel after lyophilization with scanning electron microscopy (SEM). SEM pictures show a homogenous microstructure with well-organized pores and distributed alloy microspheres were clearly visible in Mg-IHC (Figure 1E).

The above results indicate that Mg-IHC can be well shaped, ideal for filling the defect, and has a certain mechanical strength. On the other hand, it has promising degradability and desirable internal pore size, which make it suitable for nutrient transport and cell migration.

### Evaluation of the Biocompatibility of Mg-IHC

**FIGURE 2:**
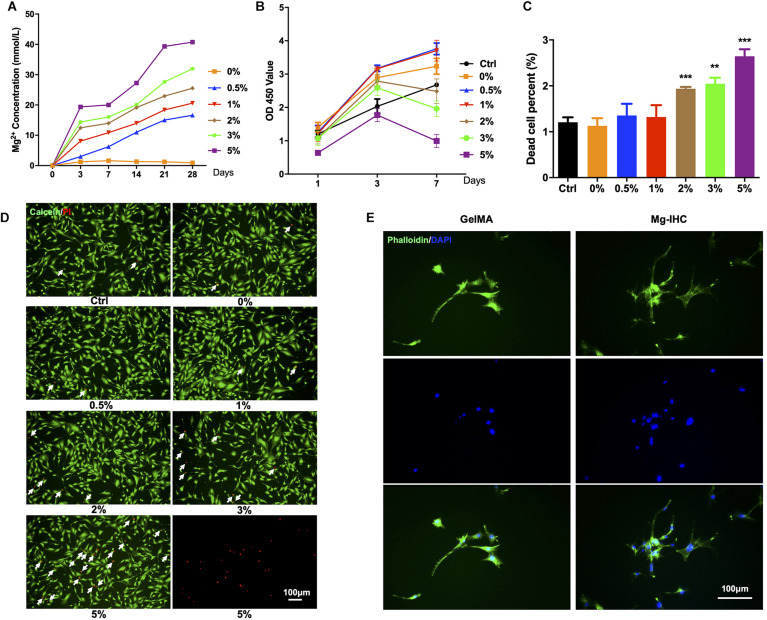
*In vitro* biocompatibility test of Mg-IHC. **(A)** Magnesium ion concentration released by Mg-IHC *in vitro* was detected after 3, 7, 14, 21, and 28 days. **(B)** Cell viability detected by CCK-8 kit on 1, 3, and 7 days, respectively. **(C)** Statistics of the percentage of dead cells. Compare with Ctrl. **P < 0.01; ***P < 0.001. **(D)** Live/dead staining for MC3T3-E1 cells. The white arrows indicate dead cells. **(E)** Cell actin cytoskeleton staining showing the morphology of MC3T3-E1 cells on different substrate, stained with FITC-phalloidin.

In order to evaluate the release of magnesium ion, Mg-IHC with different magnesium alloy contents were used to assess the concentration of magnesium ions after incubation in cell culture medium for different times. The results showed that with the increase of magnesium alloy content, the release of magnesium also increased. The concentration of magnesium also increased with time, suggesting that Mg-IHC could continuously release magnesium ion, and the increasing rate relatively slowed after 28 days (Figure 2A).

The biocompatibility is critical to the application of tissue engineering materials. Here, we evaluated cell adhesion, growth status, and proliferating activity of this material (Qiao et al., 2020). MC3T3-E1 cells were cultured with leaching medium from a series of Mg–Zn alloy proportions of GelMA. CCK-8 kit and live/dead staining were used to detect the growth status and proliferating activity. On day 1, compared with the Ctrl group, other groups showed no significant differences except the 5% group. On day 3, the value of 0.5 and 1% groups were higher than Ctrl group which had no differences from 0% (GelMA without Mg–Zn alloy), 2, 3, and 5% groups. On day 7, the value of 0, 0.5, and 1% groups were higher than ctrl, 2, 3, and 5% groups, while the 3 and 5% groups were even lower than corresponding values on day 3. All of these indicated that lower proportions (0.5 and 1%) of Mg–Zn alloy microspheres promoted the cell growth while higher proportions (2, 3, and 5%) had no similar effects (Figure 2B).

In consistence with the growth status result, Mg-IHC with lower proportions (0.5 and 1%) of Mg-Zn alloy microspheres showed no significant differences from the ctrl and 0% group in the live/dead staining. Meanwhile, the dead cell percent of Mg-IHC with higher proportions (2, 3, and 5%) were significantly higher than other groups, suggesting that lower proportions of alloy for Mg-IHC lead to higher cell viabilities (Figures 2C,D).

The cell viability decreases because of the excessive magnesium alloy content, while excessive low content also leads to the attenuated effect. Therefore, in this study, magnesium alloy hydrogels with 1% content were considered to be the most suitable. To determine the cell adhesion, we cultured cells on solidified cover layer of GelMA or Mg-IHC. Pholloidin staining results showed that cells adhered on both GelMA and Mg-IHC, and had no significant change in cell morphology (Figure 2E). Together, these results indicate that Mg-IHC has promising biocompatibility similar with GelMA *in vitro*.

To further investigate the biocompatibility of Mg-IHC, animal experiments were conducted. We examined the liver and kidney of mice with H&E staining. Compared with normal mice, the liver and kidney tissue structures of mice in GelMA and Mg-IHC groups were intact. There were no tissue edema, inflammatory cells, or tissue structure damage (Supplementary Figure S1), which indicated GelMA and Mg-IHC could be metabolized normally and had promising biocompatibility *in vivo*.

### Mg-IHC Effectiveness on Osteogenesis *in vitro*

**FIGURE 3:**
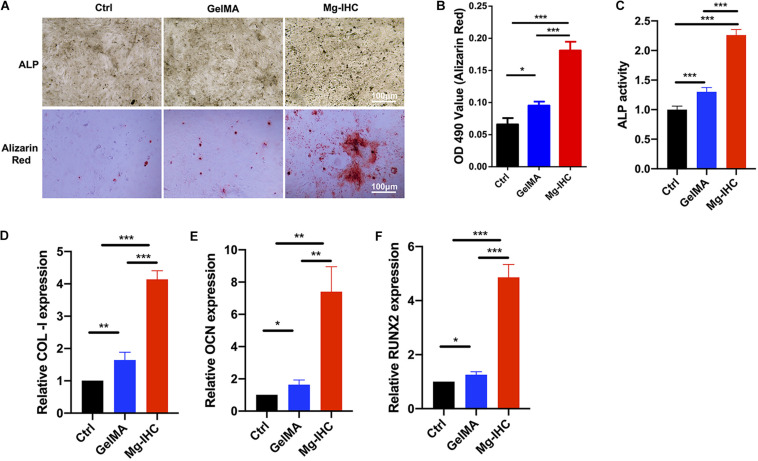
The effect of Mg-IHC on osteogenesis was examined *in vitro*. **(A)** MC3T3-E1 cells were cultured with GelMA and Mg-IHC extracts, and ALP was stained with ALP staining kit 7 days after osteogenesis induction, and calcium nodules were stained with alizarin red 14 days later. **(B)** Quantitative results of alizarin red staining. **(C)** MC3T3-E1 cells were cultured with GelMA and Mg-IHC extracts, and ALP activity was quantitatively detected using ALP activity kit 7 days after osteogenesis induction. **(D–F)** MC3T3-E1 cells were cultured with GelMA and Mg-IHC extracts, and 14 days after osteogenesis induction, the expression of osteogenesis-related genes COL-I, OCN, and RUNX2 was detected by real-time PCR. *P < 0.05; **P < 0.01; ***P < 0.001.

To further study the effects of Mg-IHC on osteogenesis, we tested with the ALP staining, activity and alizarin red staining. After 7 days’ culture and osteogenic induction, MC3T3-E1 cells were processed for ALP activity detection. Normalized with the Ctrl group, the ALP activity of GelMA group was significantly higher than the Ctrl group. Particularly, the Mg-IHC group had the highest ALP activity in the three groups. On day 14, cells were fixed for the ALP staining. In consistence with the results of ALP activity, more cells of the Mg-IHC group were labeled than the Ctrl group and the GelMA group (Figures 3A,C).

We also used alizarin red staining and quantification to assess the effect of Mg-IHC on osteogenesis. The results showed that alizarin red staining was more in the GelMA group than in the Ctrl group, while the Mg-IHC group had the strongest staining in all three groups. The results of alizarin red quantitative detection were consistent with the alizarin red staining (Figures 3A,B). Overall, these results indicated that Mg-IHC could significantly promote osteogenesis.

In addition, in order to explore the related mechanism of Mg-IHC promoting osteogenesis *in vitro*, we inspected osteogenesis-related gene indicators by real-time PCR. The results showed that the expression of collagen I (COL-I), osteocalcin (OCN), and runt-related transcription factor 2 (RUNX2) increased significantly after Mg-IHC treatment (Figures 3D–F), suggesting that Mg-IHC might promote osteogenesis by regulating the expression of COL-I, OCN, and RUNX2. COL-I is the most important organic component in bone, accounting for 85–90% of the total protein in human skeleton (Ma et al., 2014). OCN, a calcium-bound non-collagen protein secreted most by osteoblasts, is an important marker gene in the late stage of osteoblast differentiation (Nabiyouni et al., 2015). RUNX2 is a key osteogenic transcription factor and an important factor in osteoblast differentiation. ALP, a binding protein expressed in the membrane of osteoblasts, is a marker of early differentiation and maturation of osteoblasts (Lian et al., 2004). Many studies have shown that the applications of magnesium-containing materials can promote osteogenesis by promoting the expression of the genes above. Material developed by immersing magnesium ions onto the surface of micro/nanostructured titanium promotes the proliferation and differentiation of rat bone marrow mesenchymal stem cells, and also encourages the expression of OCN, ALP (Wang et al., 2014). Another magnesia-titanium material (magnesium ion incorporated titania nanotube arrays) showed a similar effect (Yan et al., 2019). The scaffolds made of magnesium hydroxyapatite nanocomposites helped the expression of ALP, COL-I, OCN, and RUNX2 in MC3T3-E1 cells with good cell adhesion and cell proliferation promoting ability (Chen et al., 2019). The biocompatible materials made with magnesium phosphate promote the gene expression of ALP, COL-I, OCN, OPN, and RUNX2, and further help the proliferation, differentiation, and mineralization of MC3T3-E cells (Nabiyouni et al., 2015). Consistent with these above studies, in our study, ALP activity detection, alizarin red staining, and quantitative detection of COL-I, OCN, and RUNX2 expression are utilized, showed that Mg-IHC could promote osteoblast proliferation as well as osteogenic differentiation and mineralization at various stages, which suggested its potential for further application *in vivo* bone repair.

### Effect of Mg-IHC in Mouse Critical-Sized Calvarial Defect

**FIGURE 4:**
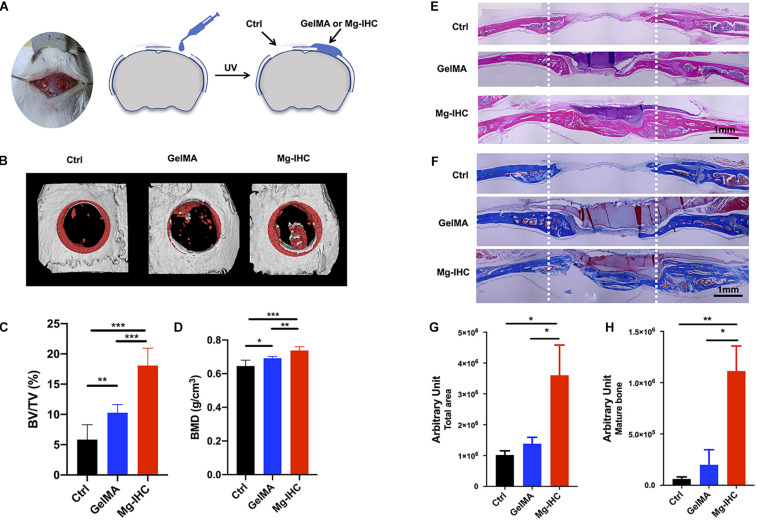
Application of Mg-IHC to the repair of critical-sized calvarial defects in mice. **(A)** Schematic illustration of Mg-IHC for calvarial defects in mice. Four weeks after the use of Mg-IHC in mouse calvarial defects. **(B)** Schematic representation of micro-CT scan reconstruction. **(C)** Quantitative results of BV/TV. **(D)** Quantitative results of BMD. **(E)** Schematic diagram of H&E staining. **(F)** Schematic diagram of Masson staining. **(G)** Quantitative results of total neoplastic tissue at the defect site. **(H)** Quantitative results of mature bone at defect site. *P < 0.05; **P < 0.01; ***P < 0.001.

To examine the bone repair effect of Mg-IHC *in vivo*, we first established a mouse model of critical-sized calvarial defect, then applied Mg-IHC composite hydrogel at the defect site, and finally carried out a series of related osteogenesis validation (Figure 4A). In previous studies, commonly used size of mouse critical-sized calvarial defect was 4 mm in diameter (Lo et al., 2012; Borrelli et al., 2019). But these are all for the sizes of single defects. Defects with diameters of 4 mm on both sides of the same skull are not appropriate, because it is inconvenient for operation. On the other hand, the defect is curved, which is not suitable for the applications of materials. It was also reported that a 3.5 mm diameter was considered as the critical-sized calvarial defect (Huang et al., 2017). And even defect with a diameter of more than 2 mm was also defined as critical size because it could not heal spontaneously within 12 weeks (Cowan et al., 2004). Therefore, in order to verify the repairing effect of Mg-IHC on bone defects, we performed a circular 3.5 mm defect on each side of the skull of mice—this size could prevent the defects to be healed by itself within 4 weeks. We filled the defects with GelMA or Mg-IHC separately to compare the results. Since the mouse skull is very thin, in order to prevent the material from detaching from the defect positions, we covered the material completely over each of the whole defects to prevent the proliferation of the tissue near the defect site which may inhibit the bone repair (Fan et al., 2018).

Micro-CT was performed to analyze the bone regeneration at the defect site. The results of three-dimensional (3D) reconstruction showed that the newly generated bone in the defect site of the GelMA group was more than that of the Ctrl group, while that of Mg-IHC group was the most significant among the three groups (Figure 4B). The quantitative results were consistent with the results of 3D reconstruction, and the ratio of BV/TV and BMD of Mg-IHC were significantly higher than those of GelMA group and Ctrl group (Figures 4C,D). These results indicated that Mg-IHC can effectively promote the repair of calvarial defects.

To further verify the effect of Mg-IHC on calvarial defect repair, we performed H&E staining and Masson staining. H&E staining result showed that there were more tissues in calvarial defects in the GelMA group than in the Ctrl group, while there were significantly more tissues in the Mg-IHC group than those of other two groups (Figure 4E). In addition, the results of Masson staining showed that, unlike the Ctrl group and the GelMA group, the overall collagen fibers as well as the mature collagen which were stained dark blue could be observed in the calvarial defects of Mg-IHC group (Figures 4F–H). All of these results suggested that Mg-IHC promoted new bone regeneration at the site of calvarial defect and improved its repair.

Compared with long bones, there are less studies focusing on magnesium and magnesium alloy-related materials in the repair of calvarial defects, which may be caused by the differences in the shape and osteogenesis mechanism of the two materials. Fixation devices made of magnesium alloys were used for cranioplasty in miniature pigs, but this study focused on biocompatibility and magnesium degradation properties, and the effect on osteogenesis was not analyzed (Naujokat et al., 2017). The preparation of biodegradable microspheres using MgO and MgCO_3_ had a certain bone regeneration effect (Yuan et al., 2019). The composite hydrogel scaffold made by bisphosphonate-magnesium can improve the adhesion and differentiation ability of mesenchymal stem cells and promote osteogenesis (Zhang et al., 2017). Different from the magnesium ion compounds commonly used in the past, in our study, atomized magnesium alloy was applied with hydrogel, which could be slowly released through the degradation of hydrogels and magnesium alloys, and thus promoted bone regeneration and osteogenesis. Unlike chemical compound of magnesium, Mg–Zn alloy degraded more moderately and would release magnesium ion constantly to promote bone regeneration. On the other hand, Mg-IHC is convenient to fabricate and can be used to fill irregular calvarial defects after injection, which grants it advantages for application. However, the use of Mg-IHC requires photo-crosslinking, but the usual UV light cannot penetrate the muscle and skin. This may lead to limited subsequent injections, if the degradation of the material is too fast while the bone defect has not been completely repaired. Therefore, the subsequent natural cross-linking solidification of multiple injectable Mg-IHC requires further research and development.

## Conclusion

In this study, we have successfully fabricated an Mg-IHC, which was prepared by mixing magnesium alloys with GelMA. We confirmed that 1% mixing ratio is suitable according to the release of magnesium ions and cell viability detection. We further confirmed continuously release of magnesium ion from Mg-IHC could improve osteogenesis by promoting the expression of osteogenesis-related genes *in vitro*. In the mouse critical-sized calvarial defect model, Mg-IHC could be injected and fixed *in situ* at the skull absorption and defect site. We observed Mg-IHC could significantly promote the bone regeneration and repair calvarial defects. All of these results indicated that the application of Mg-IHC could be an effective strategy for repairing calvarial defects in bone tissue engineering applications; and our methodology provided a convenient and effective new approach for calvarial defect repair.

## Data Availability Statement

The original contributions presented in the study are included in the article/Supplementary Material. Further inquiries can be directed to the corresponding authors.

## Ethics Statement

The animal study was reviewed and approved by the Ethics Committee of Soochow University.

## Author Contributions

W-YZ and Z-ZC conceived the study. W-YZ, Z-ZC, and QH supervised the study and edited the manuscript. W-HW designed the experiments and wrote the manuscript. W-HW, FW, and H-FZ performed the experiments and analyzed the data. KY, C-LH, and YY performed the experiments. All authors contributed to the article and approved the submitted version.

## Conflict of Interest

The authors declare that the research was conducted in the absence of any commercial or financial relationships that could be construed as a potential conflict of interest.

## Supplementary Material

Supplementary Figure 1 H&E staining of liver and kidney in normal mice, GelMA, Mg-IHC-treated mice. Blank was the sample from a normal female mouse with the same age as in GelMA and Mg-IHC groups.

## References

[B2] Agarwal S. Curtin J. Duffy B. Jaiswal S. (2016). Biodegradable magnesium alloys for orthopaedic applications: a review on corrosion, biocompatibility and surface modifications. *Mater. Sci. Eng. C Mater. Biol. Appl.* 68 948–963. 10.1016/j.msec.2016.06.020 27524097

[B3] Annabi N. Tamayol A. Uquillas J. A. Akbari M. Bertassoni L. E. Cha C. (2014). 25th anniversary article: rational design and applications of hydrogels in regenerative medicine. *Adv. Mater.* 26 85–123. 10.1002/adma.201303233 24741694 PMC3925010

[B4] Barzaghi L. R. Parisi V. Gigliotti C. R. Giudice L. Snider S. Dell’Acqua A. (2019). Bone resorption in autologous cryopreserved cranioplasty: quantitative evaluation, semiquantitative score and clinical significance. *Acta Neurochir.* 161 483–491. 10.1007/s00701-018-03789-x 30617716

[B5] Borrelli M. R. Hu M. S. Hong W. X. Oliver J. D. Duscher D. Longaker M. T. (2019). Macrophage transplantation fails to improve repair of critical-sized calvarial defects. *J. Craniofac. Surg.* 30 2640–2645. 10.1097/SCS.0000000000005797 31609958 PMC7089774

[B6] Burdette A. J. Guda T. Thompson M. E. Banas R. Sheppard F. (2018). A novel secretome biotherapeutic influences regeneration in critical size bone defects. *J. Craniofac. Surg.* 29 116–123. 10.1097/SCS.0000000000004103 29135730

[B7] Chan D. Y. C. Mok Y. T. Lam P. K. Tong C. S. W. Ng S. C. P. Sun T. F. D. (2017). Cryostored autologous skull bone for cranioplasty? A study on cranial bone flaps’ viability and microbial contamination after deep-frozen storage at -80 degrees C. *J. Clin. Neurosci.* 42 81–83. 10.1016/j.jocn.2017.04.016 28431953

[B8] Chen S. Shi Y. Zhang X. Ma J. (2019). Biomimetic synthesis of Mg-substituted hydroxyapatite nanocomposites and three-dimensional printing of composite scaffolds for bone regeneration. *J. Biomed. Mater. Res. A* 107 2512–2521. 10.1002/jbm.a.36757 31319006

[B9] Cowan C. M. Shi Y. Y. Aalami O. O. Chou Y. F. Mari C. Thomas R. (2004). Adipose-derived adult stromal cells heal critical-size mouse calvarial defects. *Nat. Biotechnol.* 22 560–567. 10.1038/nbt958 15077117

[B10] Fan M. C. Wang Q. L. Sun P. Zhan S. H. Guo P. Deng W. S. (2018). Cryopreservation of autologous cranial bone flaps for cranioplasty: a large sample retrospective study. *World Neurosurg.* 109 e853–e859. 10.1016/j.wneu.2017.10.112 29107719

[B11] Gu L. Zhang J. Li L. Du Z. Cai Q. Yang X. (2019). Hydroxyapatite nanowire composited gelatin cryogel with improved mechanical properties and cell migration for bone regeneration. *Biomed. Mater.* 14:045001. 10.1088/1748-605X/ab1583 30939454

[B12] Huang K. C. Yano F. Murahashi Y. Takano S. Kitaura Y. Chang S. H. (2017). Sandwich-type PLLA-nanosheets loaded with BMP-2 induce bone regeneration in critical-sized mouse calvarial defects. *Acta Biomater.* 59 12–20. 10.1016/j.actbio.2017.06.041 28666885

[B13] Karageorgiou V. Kaplan D. (2005). Porosity of 3D biomaterial scaffolds and osteogenesis. *Biomaterials* 26 5474–5491. 10.1016/j.biomaterials.2005.02.002 15860204

[B14] Kwon S. Lee S. S. Sivashanmugam A. Kwon J. Kim S. H. L. Noh M. Y. (2018). Bioglass-incorporated methacrylated gelatin cryogel for regeneration of bone defects. *Polymers* 10:914. 10.3390/polym10080914 30960839 PMC6403913

[B15] Li Y. Liu G. Zhai Z. Liu L. Li H. Yang K. (2014). Antibacterial properties of magnesium in vitro and in an in vivo model of implant-associated methicillin-resistant Staphylococcus aureus infection. *Antimicrob. Agents Chemother.* 58 7586–7591. 10.1128/AAC.03936-14 25288077 PMC4249557

[B16] Lian J. B. Javed A. Zaidi S. K. Lengner C. Montecino M. van Wijnen A. J. (2004). Regulatory controls for osteoblast growth and differentiation: role of Runx/Cbfa/AML factors. *Crit. Rev. Eukaryot Gene Expr.* 14 1–41. 10.1615/critreveukaryotgeneexpr.v14.i12.10 15104525

[B17] Liu Y. Chan-Park M. B. (2010). A biomimetic hydrogel based on methacrylated dextran-graft-lysine and gelatin for 3D smooth muscle cell culture. *Biomaterials* 31 1158–1170. 10.1016/j.biomaterials.2009.10.040 19897239

[B18] Lo D. D. Hyun J. S. Chung M. T. Montoro D. T. Zimmermann A. Grova M. M. (2012). Repair of a critical-sized calvarial defect model using adipose-derived stromal cells harvested from lipoaspirate. *J. Vis. Exp.* 68:4221. 10.3791/4221 23149856 PMC3499066

[B19] Lucateli R. L. Marciano M. A. Ferreira S. Garcia Junior I. R. Camilleri J. Mariano R. C. (2018). Doxycycline and autogenous bone in repair of critical-size defects. *Implant Dent.* 27 461–466. 10.1097/ID.0000000000000783 29864050

[B20] Ma J. Wang J. Ai X. Zhang S. (2014). Biomimetic self-assembly of apatite hybrid materials: from a single molecular template to bi-/multi-molecular templates. *Biotechnol. Adv.* 32 744–760. 10.1016/j.biotechadv.2013.10.014 24211471

[B21] Nabiyouni M. Ren Y. Bhaduri S. B. (2015). Magnesium substitution in the structure of orthopedic nanoparticles: a comparison between amorphous magnesium phosphates, calcium magnesium phosphates, and hydroxyapatites. *Mater. Sci. Eng. C Mater. Biol. Appl.* 52 11–17. 10.1016/j.msec.2015.03.032 25953534

[B22] Nagels J. Stokdijk M. Rozing P. M. (2003). Stress shielding and bone resorption in shoulder arthroplasty. *J. Shoulder Elbow Surg.* 12 35–39. 10.1067/mse.2003.22 12610484

[B23] Naujokat H. Seitz J. M. Acil Y. Damm T. Moller I. Gulses A. (2017). Osteosynthesis of a cranio-osteoplasty with a biodegradable magnesium plate system in miniature pigs. *Acta Biomater.* 62 434–445. 10.1016/j.actbio.2017.08.031 28844965

[B24] Pan J. Deng J. Yu L. Wang Y. Zhang W. Han X. (2019). Investigating the repair of alveolar bone defects by gelatin methacrylate hydrogels-encapsulated human periodontal ligament stem cells. *J. Mater. Sci. Mater. Med.* 31:3. 10.1007/s10856-019-6333-8 31811403

[B25] Paschoal A. L. Vanancio E. C. Canale Lde C. da Silva O. L. Huerta-Vilca D. Motheo Ade J. (2003). Metallic biomaterials TiN-coated: corrosion analysis and biocompatibility. *Artif. Organs* 27 461–464. 10.1046/j.1525-1594.2003.07241.x 12752209

[B26] Qiao Y. Liu X. Zhou X. Zhang H. Zhang W. Xiao W. (2020). Gelatin templated polypeptide co-cross-linked hydrogel for bone regeneration. *Adv. Healthc. Mater.* 9:e1901239. 10.1002/adhm.201901239 31814318

[B27] Saris N. E. Mervaala E. Karppanen H. Khawaja J. A. Lewenstam A. (2000). Magnesium. An update on physiological, clinical and analytical aspects. *Clin. Chim. Acta.* 294 1–26. 10.1016/s0009-8981(99)00258-2 10727669

[B28] Seliktar D. (2012). Designing cell-compatible hydrogels for biomedical applications. *Science* 336 1124–1128. 10.1126/science.1214804 22654050

[B29] Vega S. L. Kwon M. Y. Burdick J. A. (2017). Recent advances in hydrogels for cartilage tissue engineering. *Eur. Cell. Mater.* 33 59–75. 10.22203/eCM.v033a05 28138955 PMC5748291

[B30] Wang G. Li J. Zhang W. Xu L. Pan H. Wen J. (2014). Magnesium ion implantation on a micro/nanostructured titanium surface promotes its bioactivity and osteogenic differentiation function. *Int. J. Nanomedicine* 9 2387–2398. 10.2147/IJN.S58357 24940056 PMC4051717

[B31] Wang J. Xu J. Fu W. Cheng W. Chan K. Yung P. S. (2017). Biodegradable magnesium screws accelerate fibrous tissue mineralization at the tendon-bone insertion in anterior cruciate ligament reconstruction model of rabbit. *Sci. Rep.* 7:40369. 10.1038/srep40369 28071744 PMC5223185

[B32] Weisinger J. R. Bellorin-Font E. (1998). Magnesium and phosphorus. *Lancet* 352 391–396. 10.1016/S0140-6736(97)10535-9 9717944

[B33] Wohlrab S. Muller S. Schmidt A. Neubauer S. Kessler H. Leal-Egana A. (2012). Cell adhesion and proliferation on RGD-modified recombinant spider silk proteins. *Biomaterials* 33 6650–6659. 10.1016/j.biomaterials.2012.05.069 22727466

[B34] Wolf F. I. Cittadini A. (2003). Chemistry and biochemistry of magnesium. *Mol. Aspects Med.* 24 3–9. 10.1016/s0098-2997(02)00087-0 12537985

[B35] Yamasaki Y. Yoshida Y. Okazaki M. Shimazu A. Uchida T. Kubo T. (2002). Synthesis of functionally graded MgCO3 apatite accelerating osteoblast adhesion. *J. Biomed. Mater. Res.* 62 99–105. 10.1002/jbm.10220 12124791

[B36] Yan Y. Wei Y. Yang R. Xia L. Zhao C. Gao B. (2019). Enhanced osteogenic differentiation of bone mesenchymal stem cells on magnesium-incorporated titania nanotube arrays. *Colloids Surf. B Biointerfaces* 179 309–316. 10.1016/j.colsurfb.2019.04.013 30981066

[B37] Yu X. Shou W. Mahajan B. K. Huang X. Pan H. (2018). Materials, processes, and facile manufacturing for bioresorbable electronics: a review. *Adv. Mater.* 30:e1707624. 10.1002/adma.201707624 29736971

[B38] Yuan Z. Wei P. Huang Y. Zhang W. Chen F. Zhang X. (2019). Injectable PLGA microspheres with tunable magnesium ion release for promoting bone regeneration. *Acta Biomater.* 85 294–309. 10.1016/j.actbio.2018.12.017 30553873

[B39] Yue K. Trujillo-de Santiago G. Alvarez M. M. Tamayol A. Annabi N. Khademhosseini A. (2015). Synthesis, properties, and biomedical applications of gelatin methacryloyl (GelMA) hydrogels. *Biomaterials* 73 254–271. 10.1016/j.biomaterials.2015.08.045 26414409 PMC4610009

[B40] Zberg B. Uggowitzer P. J. Loffler J. F. (2009). MgZnCa glasses without clinically observable hydrogen evolution for biodegradable implants. *Nat. Mater.* 8 887–891. 10.1038/nmat2542 19783982

[B41] Zhang K. Lin S. Feng Q. Dong C. Yang Y. Li G. (2017). Nanocomposite hydrogels stabilized by self-assembled multivalent bisphosphonate-magnesium nanoparticles mediate sustained release of magnesium ion and promote in-situ bone regeneration. *Acta Biomater.* 64 389–400. 10.1016/j.actbio.2017.09.039 28963020

[B42] Zhu W. Wu J. Zhao H. Wang W. Lu L. Yan K. (2020). Establishment and characteristic analysis of a dog model for autologous homologous cranioplasty. *Biomed. Res. Int.* 2020:5324719. 10.1155/2020/5324719 32596324 PMC7273410

