# Supplementary figures and images for: Injectable Magnesium-Zinc Alloy Containing Hydrogel Complex for Bone Regeneration
Source: Front Bioeng Biotechnol. 2020 Nov 26;8:617585. doi: 10.3389/fbioe.2020.617585 (PMC7726114; doi:10.3389/fbioe.2020.617585)

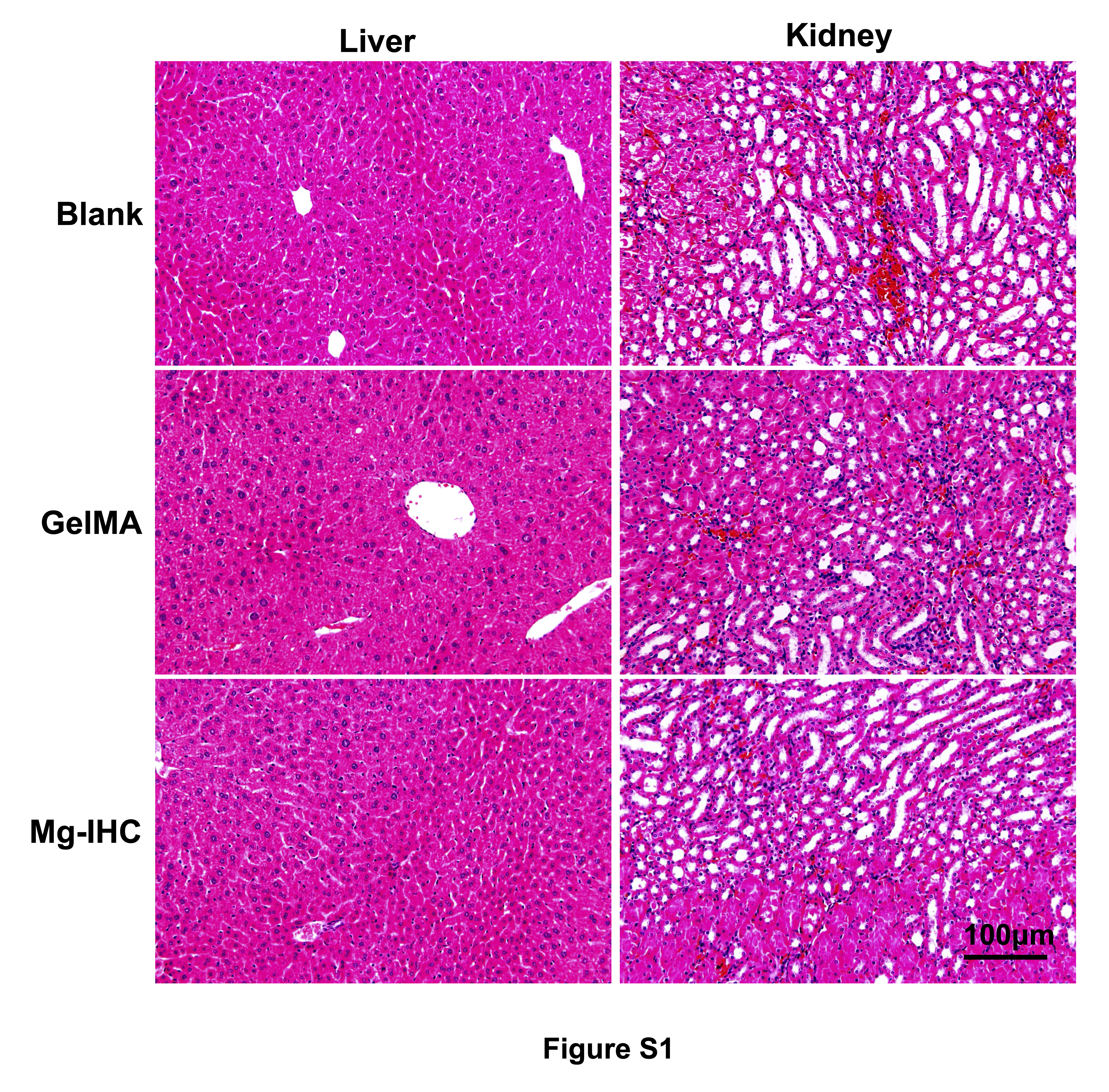

Supplement: Supplementary Figure 1 — H&E staining of liver and kidney in normal mice, GelMA, Mg-IHC-treated mice. Blank was the sample from a normal female mouse with the same age as in GelMA and Mg-IHC groups. [file Image_1.JPEG]
